# Use of giant unilamellar lipid vesicles as antioxidant carriers in in vitro culture medium of bovine embryos

**DOI:** 10.1038/s41598-022-14688-8

**Published:** 2022-07-04

**Authors:** Luana Teixeira Rodrigues Rossi, Giovana Barros Nunes, Cíntia Rodrigues da Silva, Hugo de Rossi, Priscila Helena dos Santos, Marcelo Fábio Gouveia Nogueira, Pedro Henrique Benites Aoki, Gisele Zoccal Mingoti

**Affiliations:** 1 Laboratory of Reproductive Physiology, Department of Animal Production and Health, School of Veterinary Medicine, São Paulo State University (UNESP), Clóvis Pestana Street, 793, Araçatuba Campus, São Paulo, 16050-680 Brazil; 2 Graduate Program in Veterinary Medicine, Department of Animal Reproduction, School of Agrarian and Veterinary Sciences, São Paulo State University (UNESP), Campus Jaboticabal, São Paulo, Brazil; 3 Department of Biological Sciences, School of Sciences and Languages, São Paulo State University (UNESP), Campus Assis, São Paulo, Brazil; 4 Graduate Program in Pharmacology and Biotechnology, Institute of Biosciences, São Paulo State University (UNESP), Campus Botucatu, São Paulo, Brazil; 5 Department of Biotechnology, School of Sciences and Languages, São Paulo State University (UNESP), Campus Assis, São Paulo, Brazil

**Keywords:** Animal biotechnology, Embryology

## Abstract

Giant unilamellar vesicles (GUVs) are composed of lipophilic layers and are sensitive to the action of reactive oxygen species (ROS). The use of GUVs as microcarriers of biological macromolecules is particularly interesting since ROS produced by gametes or embryos during in vitro culture can induce the opening of pores in the membrane of these vesicles and cause the release of their content. This study investigated the behavior of GUVs [composed of 2-dioleoyl-sn-glycero-3-phosphocholine and 1,2-dioleoyl-sn-glycero-3-phosphoethanolamine-*N*-(lissamine rhodamine B sulfonyl)] in co-culture with in vitro produced bovine embryos, as well as their embryotoxicity and effectiveness as cysteine carriers in culture medium. Embryonic developmental rates were unaffected, demonstrating the absence of toxicity of GUVs co-cultured with the embryos. No increase of intracellular ROS levels was observed in the embryos co-cultured with GUVs, indicating that the higher lipid content of the culture environment resulting from the lipid composition of the GUV membrane itself did not increase oxidative stress. Variations in the diameter and number of GUVs demonstrated their sensitivity to ROS produced by embryos cultured under conditions that generate oxidative stress. Encapsulation of cysteine in GUVs was found to be more effective in controlling the production of ROS in embryonic cells than direct dilution of this antioxidant in the medium. In conclusion, the use of GUVs in in vitro culture was found to be safe since these vesicles did not promote toxic effects nor did they increase intracellular ROS concentrations in the embryos. GUVs were sensitive to oxidative stress, which resulted in structural changes in response to the action of ROS. The possible slow release of cysteine into the culture medium by GUV rupture would therefore favor the gradual supply of cysteine, prolonging its presence in the medium. Thus, the main implication of the use of GUVs as cysteine microcarriers is the greater effectiveness in preventing the intracytoplasmic increase of ROS in in vitro produced bovine embryos.

## Introduction

In vitro production (IVP) of bovine embryos has grown significantly in recent decades. This biotechnology has become an important tool for accelerating the genetic improvement of cattle herds and has been applied on a large commercial scale^1, 2^.

The success of IVP is directly related to gamete quality^3^. However, the results are also influenced by extrinsic factors resulting from the excessive manipulation and exposure of gametes and embryos to the adverse conditions of the culture environment. The high concentrations of oxygen and the prolonged light exposure of these manipulated and in vitro cultured structures result in the exacerbated production of reactive oxygen species (ROS), which can reduce the developmental rates and quality of the produced embryos^4, 5^. Hydrogen peroxide (H_2_O_2_) is the most stable of all ROS and an increase in the concentration of this free radical has been previously demonstrated in in vitro cultured embryos compared to in vivo derived embryos^6^.

Reactive oxygen species are derived from various sources, including oxidative phosphorylation in the mitochondrial respiratory chain, enzymatic activation of cytochrome p450 and NADPH oxidases^7^, and these metabolites can be produced in multiple cellular compartments, including the cytosol, mitochondria, endoplasmic reticulum, peroxisomes and by plasma membrane-bound NADPH oxidase complex (NOX)^8^. The balance between the production and elimination of ROS maintains the homeostasis of the cellular reduction and oxidation (redox) processes^9^. At low concentrations, ROS participate in different physiological cell signaling pathways^10, 11^; however, the condition of oxidative stress is established when there is an imbalance between ROS production and antioxidant mechanisms. Excessive ROS production is extremely harmful to gametes and embryos since it culminates in lipid peroxidation, cellular DNA damage^12, 13^, and loss of membrane integrity as a result of oxidation, which promotes structural modifications and increases permeability of the lipid bilayer^4, 13^. In an attempt to reduce the deleterious effects caused by ROS, antioxidants are added to culture media in order to increase the antioxidant capacity of in vitro produced gametes and embryos^14–16^.

Low molecular weight thiol compounds such as cysteine have been added to oocyte maturation and embryo culture media^15, 17–19^ as precursors for the intracellular production of glutathione (GSH), a non-enzymatic antioxidant^4^ that is involved in the detoxification of lipid peroxides and removal of ROS, especially (H_2_O_2_)^20^. The biosynthesis of GSH depends on the presence of cysteine in the medium^21^, however, cysteine is unstable outside the cells and is rapidly oxidized to cystine; thus, cysteine concentrations are significantly reduced after preparation and equilibration of the medium in an incubator^22^. A previous study showed that only 40% and 3% of the initial cysteine concentration of 0.6 mM remained in the culture medium after 3 and 24 h of incubation, respectively^23^. To overcome this problem, it would be highly desirable to develop a system that permits the slow and gradual release of cysteine into the medium in order to ensure its availability for a longer period of time during culture.

Vesicles consisting of lipophilic layers were initially developed as models that mimic biological membranes^24, 25^. However, their use as potential nanotools designed to control the release of encapsulated drugs has been proposed recently^26^. These vesicles are distinguished according to the number of layers (unilamellar or multilamellar) and their size (small or liposomes, large and giant)^27^. Because of their large size (10 to 100 µm), giant unilamellar vesicles (GUVs) are particularly interesting as microcarriers of biological macromolecules^28, 29^. Additionally, GUVs are potentially interesting as oxidative stress markers during in vitro culture since they are extremely sensitive to the action of ROS^30–32^. Specifically for the use of the potential of GUVs as oxidative stress signalers in IVP cultures, it is assumed that the ROS with the greatest action would be the H_2_O_2_ produced by the cultured embryos because, unlike the other ROS, it can cross the cell membrane and diffuse for the culture medium^8^. In this context, the increased concentrations of H_2_O_2_ in the medium could act directly on the membrane of the GUVs, promoting changes in their structure with consequent release of molecules carried by them.

Despite the potential use of GUVs for molecule encapsulation^29, 30, 33, 34^, the application of these structures in reproductive biotechnologies, specifically IVP, has not yet been proposed. Therefore, this study was designed to test the hypothesis that GUVs co-cultured in vitro with bovine embryos can act as microcarriers for the delivery of the antioxidant cysteine in culture medium in the presence of oxidative stress. The aim of this study was to investigate the behavior of GUVs co-cultured with embryos, their embryotoxicity, and their effectiveness as cysteine carriers.

## Results

### GUVs cultured in vitro in the absence of cells are not affected by the oxidizing effect of menadione

**Figure 1:**
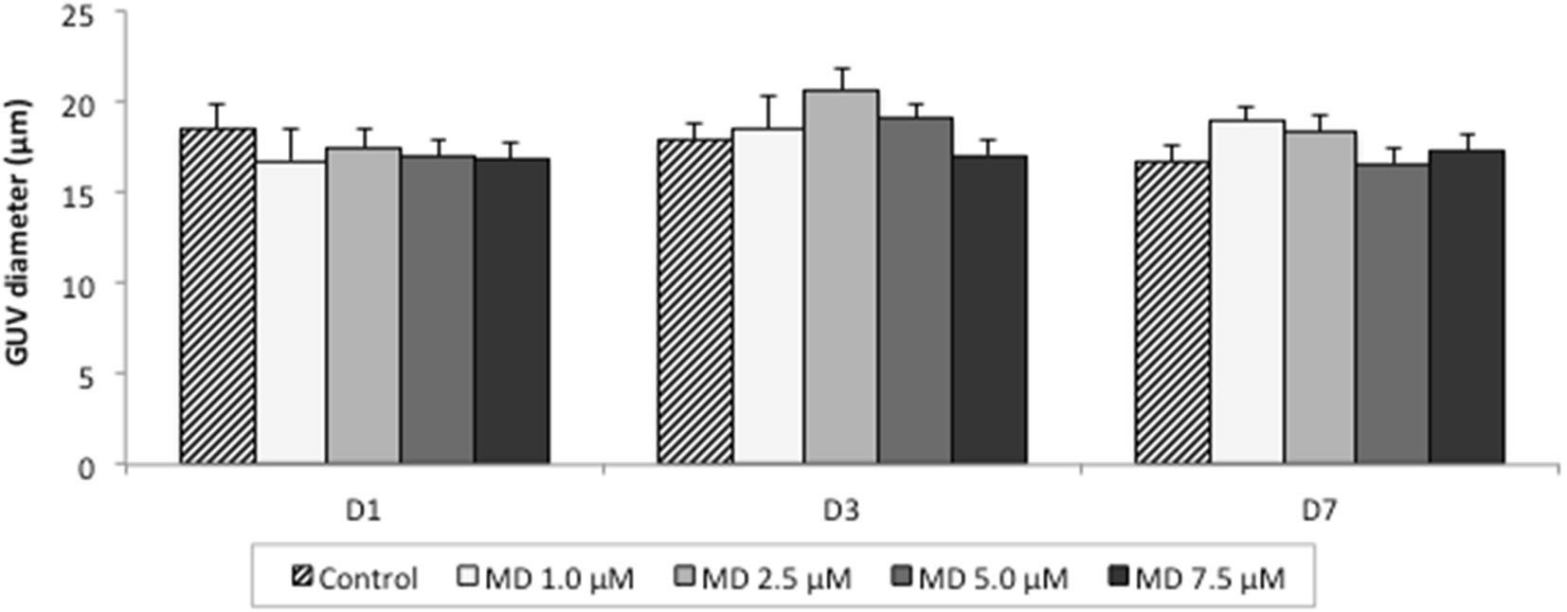
Diameter of giant unilamellar vesicles (GUVs) cultured in vitro with different concentrations of menadione (MD). Data are expressed as the mean ± standard error of three independent replicates (control, *n* = 147; MD 1.0 µM, *n* = 119; MD 2.5 µM, *n* = 134; MD 5.0 µM, *n* = 132; and MD 7.5 µM, *n* = 148). *D1 = day 1 (beginning of cultivation); D3 = day 3 (72-h cultivation); D7 = day 7 (168-h cultivation). No differences were observed between treatments within each assessment day (*P* > 0.05).

Menadione (MD) is a compound used to induce oxidative stress due to its capacity to trigger the production of superoxide anion (O_2_^−^), H_2_O_2_ and other ROS in cells cultured in the presence of oxygen^30^. ROS can cause structural modifications in GUVs, especially alterations in their diameter^33, 35–38^. The variation in GUV diameter was therefore used as a parameter to evaluate menadione-induced oxidative stress in the in vitro culture system in the absence of cells. The diameter of GUVs cultured at different concentrations of menadione (Fig. 1) was similar between treatments at the beginning of culture (day 1—D1; *P* > 0.05), after 72 h (D3; *P* > 0.05) and at the end of culture (D7; *P* > 0.05). There was no difference between any of the concentrations tested and the control group (*P* > 0.05).

### The morphometry of GUVs cultured in vitro in the absence of cells is affected by hydrogen peroxide

**Figure 2:**
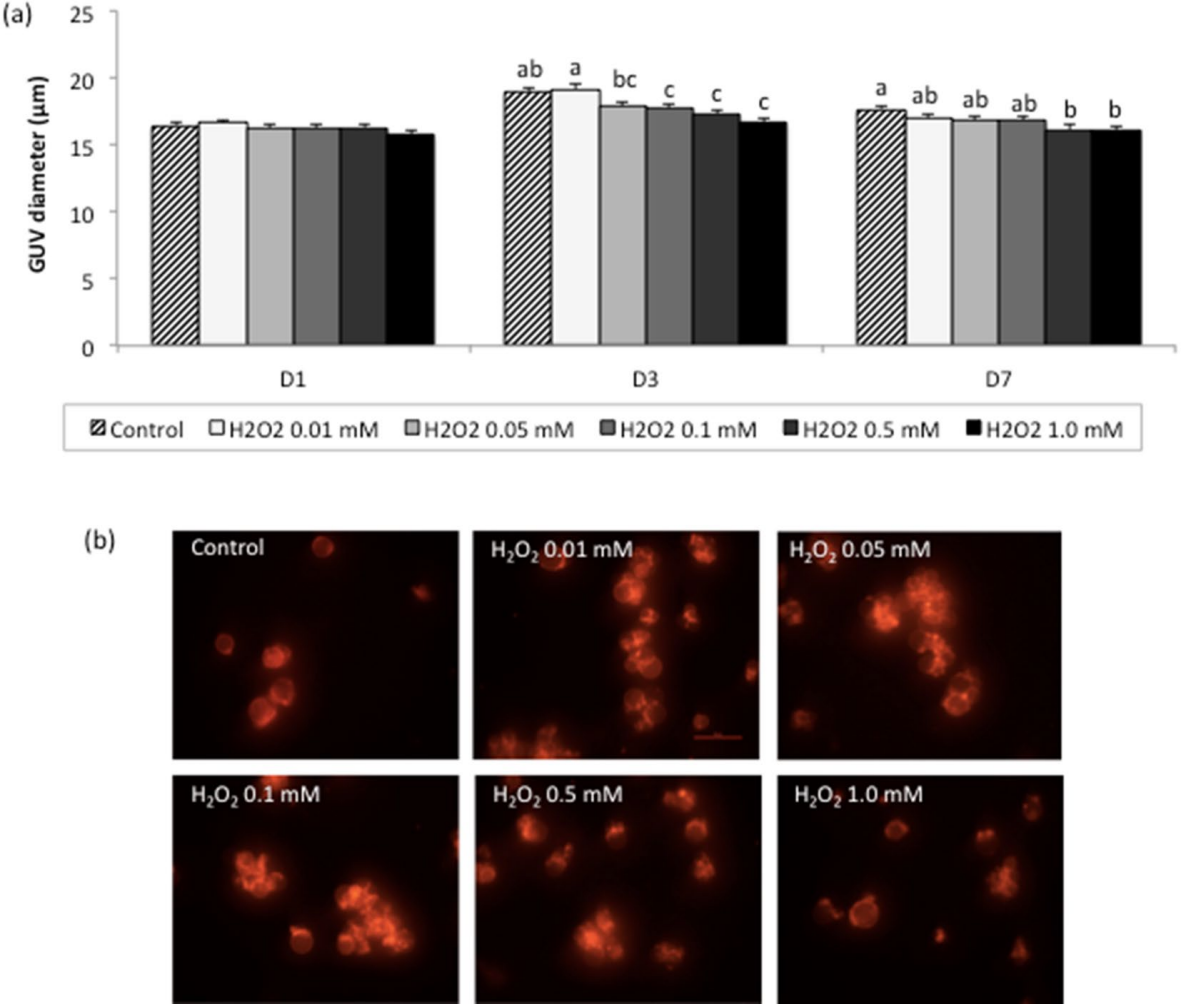
Diameter of giant unilamellar vesicles (GUVs) cultured in vitro with different concentrations of hydrogen peroxide (H_2_O_2_). (**a**) Data are expressed as the mean ± standard error of three independent replicates (control, *n* = 1,031; H_2_O_2_ 0.01 mM, *n* = 891; H_2_O_2_ 0.05 mM, *n* = 882; H_2_O_2_ 0.1 mM, *n* = 786; H_2_O_2_ 0.5 mM, *n* = 907; and H_2_O_2_ 1.0 mM, *n* = 698). *D1 = day 1 (beginning of cultivation); D3 = day 3 (72-h cultivation); D7 = day 7 (168-h cultivation). Different letters within each assessment day indicate significant differences (*P* < 0.05). (**b**) Representative photomicrographs of GUVs collected from culture wells on D7 of the different treatments. ×40 Magnification.

In this experiment we evaluated the alterations in the diameter of in vitro-cultured GUVs after stimulation with H_2_O_2_, one of the main and most potent free radicals produced by cells in response to oxidative stress^4^. The diameter of GUVs cultured with H_2_O_2_ in the absence of cells (Fig. 2a) was similar between treatments at the beginning of culture (D1; *P* > 0.05). However, on D3, a reduction (*P* < 0.05) in diameter was observed in GUVs cultured in the presence of the higher concentrations of H_2_O_2_ tested (0.1 mM: 17.71 ± 0.30 µm; 0.5 mM: 17.32 ± 0.29 µm; 1.0 mM: 16.63 ± 0.31 µm) when compared to control (18.96 ± 0.27 µm). Likewise, the diameter of GUVs cultured in the presence of the higher concentrations of H_2_O_2_ tested (0.5 mM: 16.14 ± 0.36 µm; 1.0 mM: 16.13 ± 0.31 µm) was reduced at the end of culture (D7) when compared to control (17.67 ± 0.30 µm). Representative photomicrographs of GUVs cultured for 7 days with or without H_2_O_2_ are shown in Fig. 2b.

### GUVs are not embryotoxic but are sensitive to oxidative stress induced by menadione in in vitro cultured embryos

In the first series of this experiment, we demonstrated the induction of oxidative stress in cultured embryos exposed to menadione. Next, we evaluated (1) the embryotoxicity of GUVs; (2) intracytoplasmic concentrations of ROS in embryos co-cultured with GUVs, and (3) the morphometry and quantity of GUVs co-cultured with embryos subjected to menadione-induced oxidative stress.

**Figure 3:**
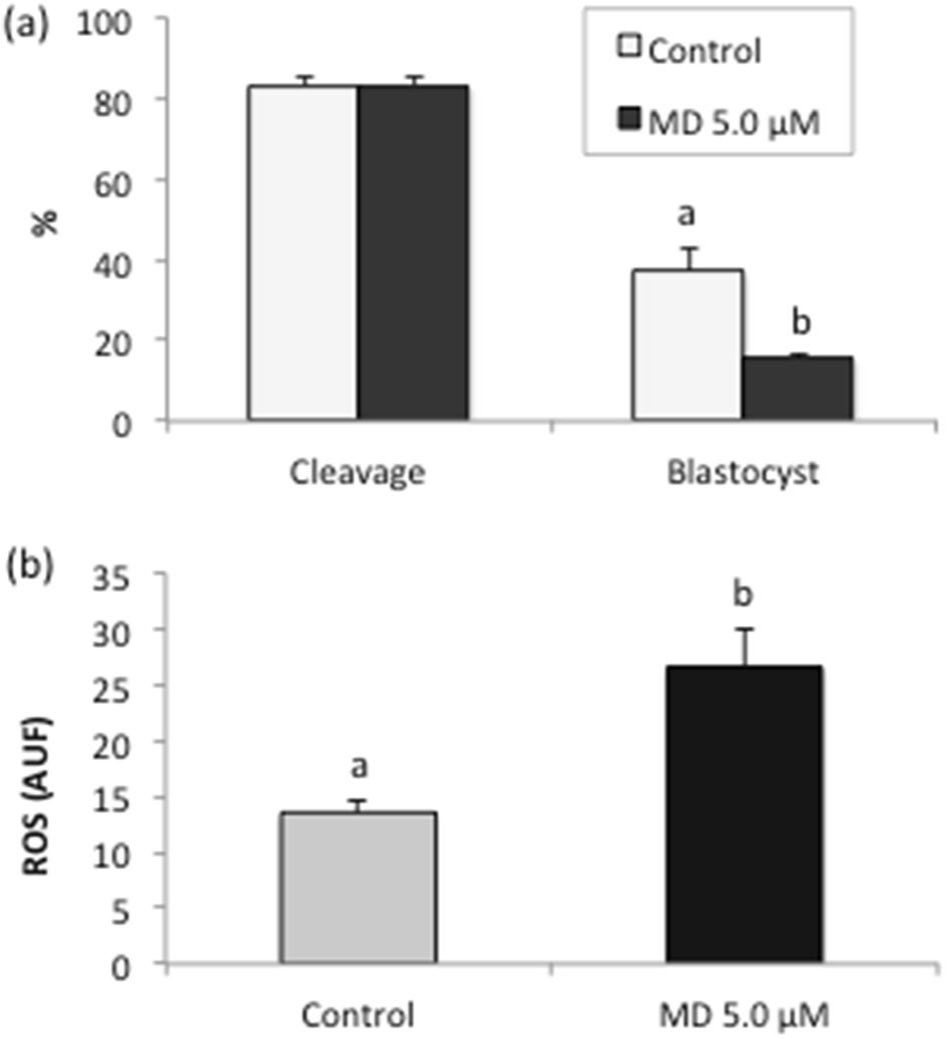
Cleavage and development (**a**) and intracellular concentrations of reactive oxygen species (ROS) (**b**) of bovine embryos cultured in vitro and subjected to menadione (MD)-induced oxidative stress. Data are expressed as the mean ± standard error of four independent replicates. Embryos were cultured for 7 days, and menadione (5.0 μM) was included in the medium only on the sixth day of culture (from D6 to D7).

The effects of supplementation with menadione during embryo culture on embryo outputs and generation of oxidative stress are given in Fig. 3. No difference (*P* > 0.05) in the cleavage rate was observed between treatments, but blastocyst rate was decreased in the presence of menadione (control group: 37.24% ± 5.4 vs. MD 5.0 μM group: 15.75% ± 0.9; *P* < 0.05, Fig. 3a). Higher intracellular ROS concentrations (Fig. 3b) were observed in embryos of the MD 5.0 μM group (26.61 ± 3.29 AUF) when compared to the control (13.56 ± 0.99 AUF; *P* < 0.05).

No difference (*P* > 0.05) in the cleavage rate was observed between treatments (Table 1). There was no difference (*P* > 0.05) in the embryonic developmental rate up to the blastocyst stage between control (26.70% ± 7.30) and GUV groups (25.18% ± 4.32), but both groups exhibited higher blastocyst rates (*P* < 0.05) than the groups treated with different concentrations of menadione (GUV + MD 5.0 μM: 6.95% ± 3.13 and GUV + MD 7.5 μM: 0.95% ± 0.95).

**Table 1:** Development and intracellular concentrations of reactive oxygen species (ROS) in bovine embryos cultured in vitro in the presence of giant unilamellar vesicles (GUVs) and subjected to menadione (MD)-induced oxidative stress.

Groups	Oocytes (*n*)	Embryo development		ROS levels in D7 blastocysts
		Cleavage (%)	Blastocysts (%)	
Control	100	86.35 ± 3.38	26.70 ± 7.30^a^	3.76 ± 0.46^a^
GUV	104	89.54 ± 1.43	25.18 ± 4.32^a^	3.99 ± 0.33^a^
GUV + MD 5.0 μM*	104	89.75 ± 2.69	6.95 ± 3.13^b^	16.28 ± 4.51^b^
GUV + MD 7.5 μM	100	92.95 ± 2.99	0.95 ± 0.95^b^	–

Data are expressed as the mean ± standard error of four independent replicates.

*Embryos were cultured for 7 days (D7), and menadione (5.0 μM or 7.5 μM) was included in the medium only on the sixth day of culture (from D6 to D7). ROS concentrations are expressed as arbitrary units of fluorescence (AUF).

^ab^Different letters in each column indicate significant differences (*P* < 0.05).

Higher intracellular ROS concentrations (Table 1) were observed in embryos of the GUV + MD 5.0 μM group (16.28 ± 4.61 AUF), which differed (*P* < 0.05) from the control (3.76 ± 0.46 AUF) and GUV (3.99 ± 0.33 AUF) groups. Embryos of the GUV + MD 7.5 μM group were not evaluated because of the lack of development of an adequate number of structures for this assessment.

There was no difference in the diameter of GUVs between the different treatments at the beginning of culture (D1; *P* > 0.05) (Table 2). However, an increase (*P* < 0.05) in GUV diameter was observed at the end of culture (D7) in the GUV + MD 7.5 μM group (17.79 ± 0.94 µm) compared to the GUV (14.63 ± 0.82 µm) and GUV + MD 5.0 μM (13.49 ± 0.59 µm) groups. There was also a variation overtime (D1 vs. D7) in the diameter of GUVs cultured in the presence of menadione (*P* > 0.05).

**Table 2:** Effect of menadione (MD) on the diameter of giant unilamellar vesicles (GUVs) co-cultured in vitro with bovine embryos.

Groups	GUV diameter on D1^#^		GUV diameter on D7	
	*n*	Diameter (μm)	*n*	Diameter (μm)
GUV	843	14.92 ± 0.19	77	14.63 ± 0.82^b^
GUV + MD 5.0 μM^##^	708	14.90 ± 0.18	86	13.49 ± 0.59^b^*
GUV + MD 7.5 μM	790	15.30 ± 0.19	69	17.79 ± 0.94^a^*

Data are expressed as the mean ± standard error of four independent replicates.

^#^D1 = day 1 (beginning of cultivation); D7 = day 7 (end of 168-h cultivation).

^##^Embryos were cultured for 7 days, and menadione (5.0 μM or 7.5 μM) was included in the medium only on the sixth day of culture (from D6 to D7).

^ab^Different letters in each column indicate significant differences (*P* < 0.05). The asterisk (in the same row) indicates a significant difference within the same treatment when evaluated on different days (*P* < 0.05).

**Figure 4:**
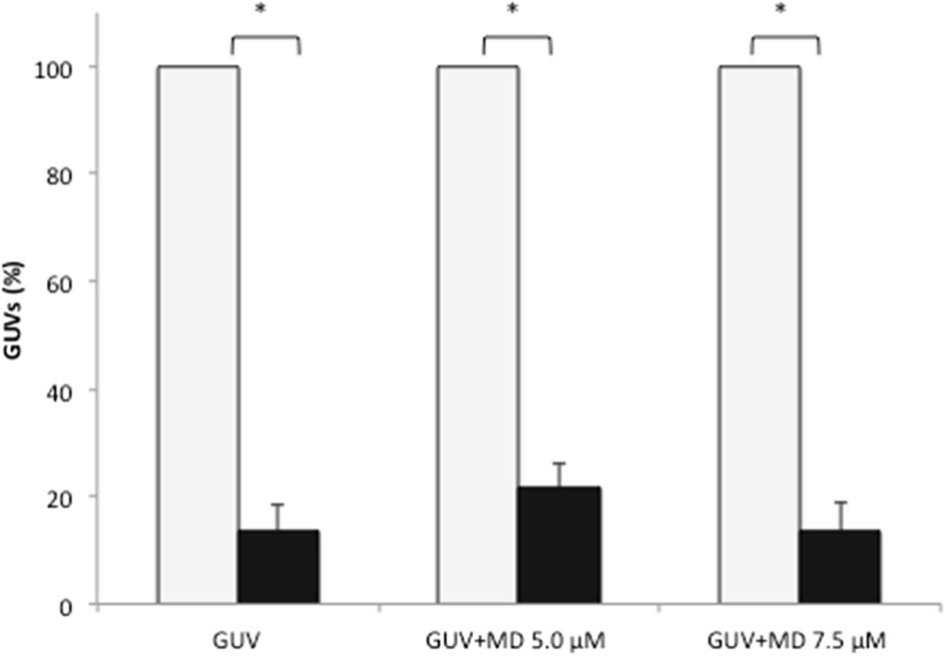
Percentage of giant unilamellar vesicles (GUVs) present in droplets of in vitro cultured bovine embryos. The percentage of GUVs on the last day of culture (D7: black bars) was determined in relation to the total number of GUVs present on the first day (D1: light gray bars). On D6, oxidative stress was induced in the embryos by the addition of different concentrations of menadione (MD) for 24 h. There was no significant difference between treatments at each time point (*P* > 0.05). The asterisk indicates a significant difference within the same treatment when evaluated on different days (*P* < 0.05).

No difference between treatments (*P* > 0.05) was found for the variation in the number of GUVs (%) present in the wells at the end of culture (D7) compared to D1, with 13.6% ± 4.9 of remnant GUVs on D7 in the GUV group, 21.6% ± 4.7 in the GUV + MD 5.0 μM group, and 13.5% ± 5.3 in the GUV + MD 7.5 μM group (Fig. 4). However, the number of remnant GUVs in the wells was decreased overtime (D1 vs. D7) in all groups (*P* < 0.05).

### Supplementation of in vitro culture medium with antioxidant encapsulated in GUVs provides greater protection against embryonic oxidative stress than supplementation through direct dilution in the medium

The suitability of GUVs as antioxidant carriers in embryo culture medium was evaluated in this experiment. For this purpose, the effect of the antioxidant cysteine diluted in culture medium [concentration of 0.6 mM as recommended in previous studies^14, 15^ was compared to that of cysteine (Cyst) encapsulated in GUVs [GUV(Cyst 3 mM)]. These GUVs were obtained by electroformation in a solution containing 3 mM cysteine (5X solution) and subsequent dilution in the culture medium (20%, v/v) in order to obtain a maximum final concentration of cysteine in the medium of 0.6 mM after complete content release from the GUVs.

The cleavage rate was similar (*P* > 0.05) between treatments (Table 3). The rate of embryo development to the blastocyst stage was lower (*P* < 0.05) in the groups subjected to menadione-induced oxidative stress (MD: 9.46% ± 6.04; Cyst + MD: 1.19% ± 1.19; GUV(Cyst 3 mM) + MD: 1.15% ± 1.15) compared to the control (25.58% ± 4.53), Cyst (31.77% ± 0.37), and GUV(Cyst 3 mM) (30.16% ± 1.59) groups.

**Table 3:** Cleavage and development of bovine embryos cultured in vitro in medium supplemented with giant unilamellar vesicles (GUVs) and cysteine (Cyst) and subjected to menadione (MD)-induced oxidative stress.

Groups	Culture medium additive		Oocytes (*n*)	Embryo development (%)	
	MD^A^	Cysteine		Cleavage	Blastocysts
Control	−	−	111	87.99 ± 0.92^a^	25.58 ± 4.53^a^
Cyst	−	0.6 mM (diluted in medium)^B^	112	84.65 ± 4.83^a^	31.77 ± 0.37^a^
GUV(Cyst 3 mM)	−	3 mM (in GUV)^C^	116	83.97 ± 5.71^a^	30.16 ± 1.59^a^
MD	+	−	111	91.69 ± 1.04^a^	9.46 ± 6.04^b^
Cyst + MD	+	0.6 mM (diluted in medium)	110	90.01 ± 1.56^a^	1.19 ± 1.19^b^
GUV(Cyst 3 mM) + MD	+	3 mM (in GUV)	113	88.37 ± 4.01^a^	1.15 ± 1.15^b^

Data are expressed as the mean ± standard error of four independent replicates.

^A^Embryos were cultured for 7 days, and menadione (5.0 μM) was included in the medium only on the sixth day of culture (from D6 to D7).

^B^In the Cyst and Cyst + MD groups, cysteine was diluted directly into the culture medium at the final concentration of 0.6 mM.

^C^In the GUV(Cyst 3 mM) and GUV(Cyst 3 mM) + MD groups, the GUVs were electroformed in a solution containing 3 mM cysteine (5X solution) and subsequently diluted in the culture medium (20% v/v) in order to obtain a maximum final concentration of cysteine in the medium of 0.6 mM after complete content release from the GUVs.

^ab^Different letters in each column indicate significant differences (*P* < 0.05).

**Figure 5:**
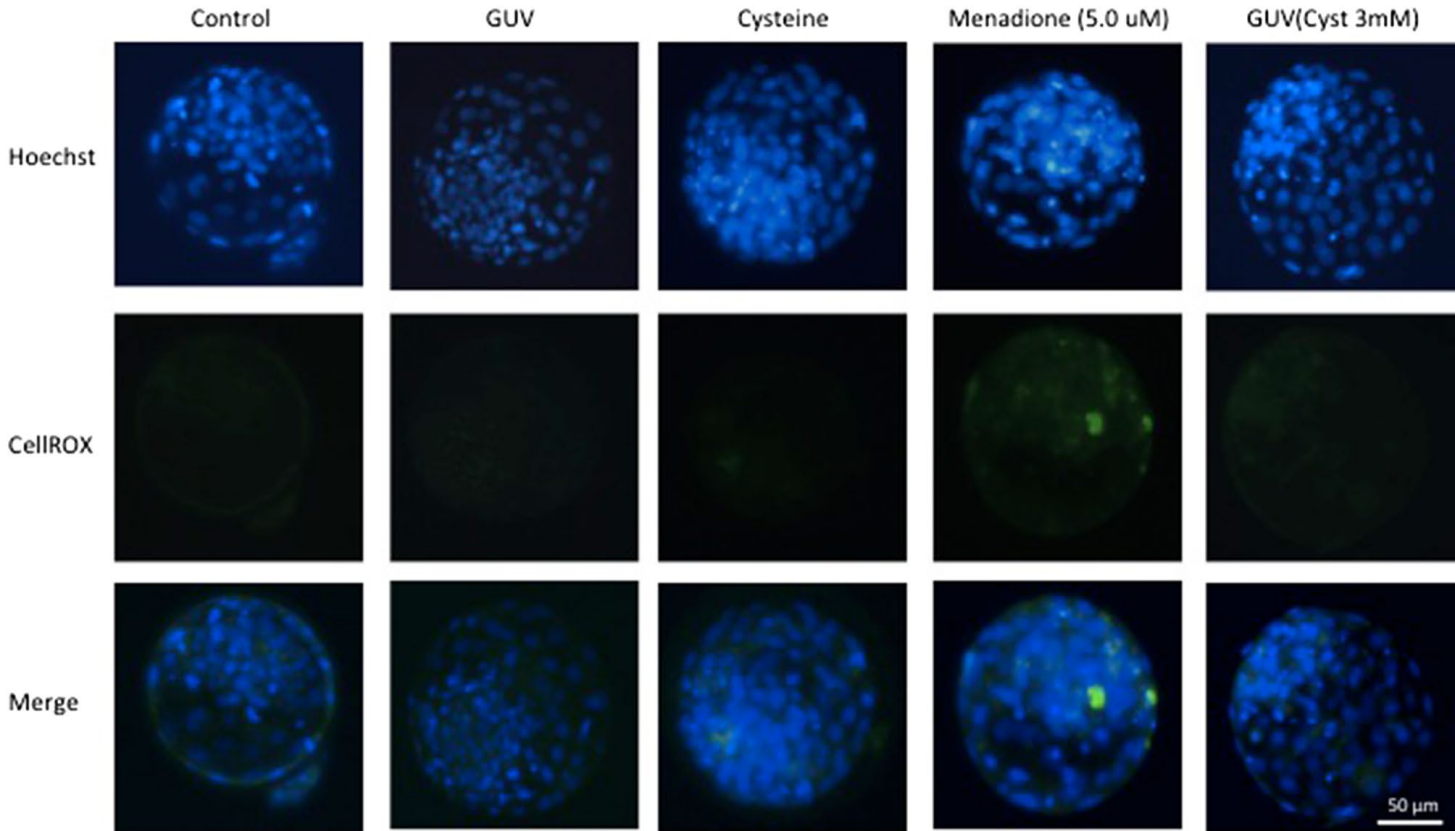
Representative photomicrographs of embryos from the different treatments stained for reactive oxygen species detection (CellROX Green Reagent = green) and DNA (Hoechst = blue).

Intracellular concentrations of ROS were evaluated in embryos on D7 (Table 4), except for the Cyst + MD and GUV(Cyst) + MD groups because of the lack of development of an adequate number of structures for this assessment. Embryos of the GUV(Cyst 3 mM) group had lower (*P* < 0.05) ROS concentrations (0.09 ± 0.01 AUF) than those of the Cyst group (0.16 ± 0.02 AUF), but neither group differed from the control group (0.10 ± 0.01 AUF). The highest ROS concentrations were observed in the MD group (0.34 ± 0.05 AUF), which differed from the other treatments (*P* < 0.05). Representative photomicrographs of embryos from the different treatments stained for reactive oxygen species are shown in Fig. 5.

**Table 4:** Intracellular concentrations of reactive oxygen species (ROS) in bovine embryos cultured in vitro in medium supplemented with giant unilamellar vesicles (GUVs) and cysteine (Cyst) and subjected to menadione (MD)-induced oxidative stress.

Groups	Culture medium additive		Embryos (*n*)	ROS (AUF)
	MD^A^	Cysteine		
Control	−	−	21	0.10 ± 0.01^bc^
Cyst	−	0.6 mM (diluted in medium)^B^	29	0.16 ± 0.02^b^
GUV(Cyst 3 mM)	−	3 mM (in GUV)^C^	25	0.09 ± 0.01^c^
MD	+	−	9	0.34 ± 0.05^a^

Data are expressed as the mean ± standard error of four independent replicates.

^A^Embryos were cultured for 7 days, and menadione (5.0 μM or 7.5 μM) was included in the medium only on the sixth day of culture (from D6 to D7).

^B^In the Cyst group, cysteine was diluted directly into the culture medium at the final concentration of 0.6 mM.

^C^In the GUV(Cyst 3 mM) group, the GUVs were electroformed in a solution containing 3 mM cysteine (5X solution) and subsequently diluted in the culture medium (20% v/v) in order to obtain a maximum final concentration of cysteine in the medium of 0.6 mM after complete content release from the GUVs. ROS concentrations are expressed as arbitrary units of fluorescence (AUF).

^abc^Different letters in the column indicate significant differences (*P* < 0.05).

## Discussion

This is the first study that proposes the use of GUVs as antioxidant carriers for supplementation of in vitro culture media of bovine embryos. Overall, the results showed that GUVs are not embryotoxic and are sensitive to ROS produced by embryos cultured under conditions that generate oxidative stress. Additionally, encapsulation of the antioxidant in GUVs was found to be more effective in controlling the production of ROS in embryonic cells than direct dilution of the antioxidant in the medium.

The present results revealed no changes in the diameter of GUVs cultured in vitro in the presence of menadione. This compound has been used in previous studies to induce oxidative stress in in vitro cultured cells^39^. It acts by disrupting the cellular pyridine nucleotide redox balance, compromising cellular ATP production and mitochondrial respiratory activity^40^. As a consequence, cells produce the main ROS such as O_2_^−^ and H_2_O_2_^41^. Although we did not investigate the presence of ROS in the culture medium, the results suggest that menadione did not induce the generation of ROS because of the absence of cells; thus, this drug did not directly trigger oxidative stress in GUVs. In a second step, we evaluated the direct effect of a free radical (H_2_O_2_) on GUVs. This radical promoted changes in the diameter of these structures even when cultured in vitro in the absence of cells. This variation in surface area and the concomitant morphological changes in GUVs have been previously observed in response to irradiation in the presence of photooxidative substances^36^.

After determination of the sensitivity of GUVs to H_2_O_2_, these structures were co-cultured with embryos under conditions that generate oxidative stress (menadione challenge) in order to evaluate the susceptibility of GUVs to ROS produced by cultured cells. Variations in the diameter and number of remnant GUVs were observed at the end of the culture period in the group treated with the highest menadione concentration tested (7.5 µM). This result corroborates the sensitivity of GUVs to H_2_O_2_ and might be explained by the fact that these vesicles, which are composed of unsaturated phospholipids, are extremely sensitive to lipid peroxidation, a chain reaction mediated by oxidative products^42^. Recent studies have shown that the exposure of GUVs to photooxidative substances leads to the cleavage of lipid chains and the consequent opening of pores in the membrane, causing changes in its structure^33, 43, 44^ such as surface area variation, associated or not with membrane fluctuations and the release of “buds” due to oxidative stress^36, 37, 42^. Indeed, it has previously been reported that ROS can react with the unsaturations of the lipid chains forming hydroperoxides. The hydrophilic nature of these groups favors migration towards the polar heads of the lipids leading to an increase of the mean molecular area per lipid and the overall surface area expansion of the GUVs^33, 45^. In addition, the propagation of the oxidation reactions may lead to the chain cleavage at the unsaturation sites, inducing the pore opening in the lipid membrane^33, 46^ and further decrease of the GUV dimension. Although small, the reduction in population of GUVs over time is indeed significant. This effect is inherent to the stability of the colloidal system formed by self-assembled lipid structures^47^, which can be attributed to a variety of environmental conditions, such as temperature and ionic strength. Thus, our results thus suggest that the variation in the diameter of GUVs may be the result of their sensitivity to oxidative stress. Although we did not evaluate the ultrastructure of GUVs, literature data indicate that the variation in GUV diameter may be a consequence of membrane damage caused by ROS, especially by the H_2_O_2_ radical^33, 35–38, 43, 44^. One may thus assume that this membrane damage promotes possible extravasation of the content of GUVs.

Since GUVs are considered useful tools for macromolecule encapsulation^29^ and free radicals can damage their membrane^37^, causing possible extravasation of their content, these structures are excellent candidate vehicles for the gradual release of antioxidants into the in vitro culture medium of embryos in situations that generate oxidative stress. Within this context, the supply of antioxidant to the medium via GUVs would permit a response to the specific needs of the culture system since the unnecessary or excessive inclusion of antioxidants can be harmful^19^. For example, at low concentrations, ROS play a physiological role in cells; more specifically, they regulate cell function by controlling the production and activation of substances with biological activity in the main signaling pathways^48, 49^. To our knowledge, there are no studies that have co-cultured GUVs with embryos. Thus, their embryotoxicity needs to be evaluated. The present results demonstrated that GUVs did not affect embryonic development and do therefore not exert any morphologically evident embryotoxic effect. Likewise, no increase of intracellular ROS levels was observed in embryos cultured in vitro in the presence of GUVs, indicating that the higher lipid content of the culture environment resulting from the lipid composition of the GUV membrane itself did not cause any increase in oxidative stress. Based on these satisfactory results, cysteine was encapsulated in GUVs by electroformation since the presence of cysteine in the culture medium is essential for the synthesis of GSH by the embryo^14^, favoring the quality of the quality of the embryos produced. However, supplementation of the culture medium with cysteine, either diluted or encapsulated in GUVs, did not promote an increase in blastocyst production rates when the cultures were not challenged with menadione, although an interesting numerical increase in embryo production was observed [25.58% in the control group vs. 31.77% and 30.16% in the Cyst and GUV(Cyst 3 mM) groups, respectively]. Similarly, in the absence of oxidative stress, neither cysteine supplementation system reduced intracytoplasmic ROS levels in the embryos compared to control. According to the literature, the addition of antioxidants during in vitro culture produces contradictory results in terms of embryo development, with studies reporting beneficial^14^, undesirable^50^, or no effects^51^. It is most likely that beneficial effects are only seen when the culture conditions are inadequate.^52^ Thus, the lack of effects of the antioxidant suggests that the culture conditions in this study were satisfactory and did not generate excessive oxidative stress.^48, 49^

In the present study, although supplementation of the medium with 0.6 mM cysteine did not compromise embryo developmental rates, the results showed that supplementation with this antioxidant encapsulated in GUVs was more effective than its direct dilution in the medium in preventing the increase of intracytoplasmic ROS levels in embryos caused by the inherent conditions of the culture system itself, i.e., in the absence of menadione [0.09 AUF in the GUV (Cyst 3 mM) vs. 0.16 AUF in the Cyst group]. One may speculate that the direct dilution of cysteine in the culture medium may have resulted in its rapid oxidation into cystine^53, 54^, consequently reducing the availability of the former as precursors of GSH synthesis^17, 53^. On the other hand, the possible slow release of cysteine into the culture medium by rupture of the GUVs would favor the gradual supply of cysteine, prolonging its presence in the medium. These results are promising and encourage the continuation of this study in order to implement this tool in reproductive biotechnology protocols.^17, 53^

In summary, we demonstrated that in vitro cultured GUVs had no toxic effect and did not increase intracellular ROS concentrations in the embryos. The use of these structures was safe for the IVP purposes described in this study. Since they are sensitive to oxidative stress, GUVs underwent structural changes in response to the action of ROS, indicating that these structures are suitable microcarriers for the on-demand release of antioxidants. Compared to the system in which cysteine was diluted directly in the medium (without encapsulation), GUVs containing cysteine were more effective in preventing the intracytoplasmic increase of ROS in in vitro produced bovine embryos. These results indicate that the use of GUVs as microcarriers is safe and beneficial for improving the quality of in vitro produced embryos. The findings should encourage new studies implementing the use of this support tool in order to improve the efficiency of reproductive biotechnologies.

## Methods

### Ethics approval

All experimental procedures were approved by the Ethics Committee on Animal Experimentation of UNESP (Protocol No. 009981/17) and were conducted in accordance with relevant guidelines and regulations of São Paulo State University, Campus of Araçatuba and Campus of Assis, Brazil. All methods used in this study are reported in accordance with ARRIVE guidelines for the reporting of animal experiments.

### Chemical reagents and media

The reagents were purchased from Sigma-Aldrich (St Louis, MO, USA), unless otherwise stated. Plastic materials and tubes were obtained from Corning Inc. (Acton, MA, USA).

The in vitro maturation (IVM) medium consisted of TCM199 supplemented with 10% (v/v) fetal calf serum (FCS; Gibco BRL, Grand Island, NY, USA), 0.2 mM sodium pyruvate, 25 mM sodium bicarbonate, 50 μg/mL amikacin, 0.5 μg/mL FSH (Folltropin-V; Bioniche Animal Health, Ontario, Canada), and 100 IU/mL hCG (Vetecor; Hertape Calier, Juatuba, MG, Brazil). The fertilization medium (IVF-TALP) consisted of Tyrode’s albumin lactate pyruvate (TALP) containing 0.2 mM Na-pyruvate, 6 mg/mL fraction V fatty acid–free BSA, 25 mM sodium bicarbonate, 13 mM Na-lactate, 50 μg/mL amikacin, 10 μM hypotaurine, 20 μM penicillamine, 2 μM epinephrine, and 10 μg/mL heparin. The embryo culture medium contained modified synthetic oviductal fluid (mSOF) supplemented with 0.2 mM L-glutamine, 0.34 mM sodium citrate, 2.8 mM myo-inositol, 2% essential amino acids, 1% non-essential amino acids, 0.2 mM pyruvate, 50 μg/mL amikacin, 5 mg/mL BSA, and 2.5% (v/v) FCS.

Cysteine (Sigma C7352) was freshly diluted in mSOF daily during the experiment to obtain a final concentration of 0.6 mM^14, 15^.

### Electroformation and evaluation of giant unilamellar vesicles

The GUVs were prepared by electroformation^33^, with some modifications^55^. Phospholipids 2-dioleoyl-sn-glycero-3-phosphocholine (DOPC) and 1,2-dioleoyl-sn-glycero-3-phosphoethanolamine-*N*-(lissamine rhodamine B sulfonyl) (Liss Rod PE) were purchased from Avanti Polar Lipids, Inc. (Alabaster, AL, USA). DOPC GUVs were diluted in chloroform at a concentration of 10^−3^ mol/L. Fluorescent GUVs were obtained by adding 0.5 mol% of Liss Rod PE. Next, 10 μL of the chloroform solution containing the lipid mixture was spread on the surfaces of two conductive Indium tin oxide-coated slides, which were then assembled to form a growing chamber separated by a 2-mm-thick Teflon frame. After drying under vacuum, the chamber was filled with 260 mOsm kg^−1^ sucrose solution that contained 3 × 10^−3^ mol/L cysteine [GUV (Cyst 3 mM)] or not [GUV]. The osmolarity of the sucrose solution was set to match the osmolarity of the mSOF medium (260 mOsm kg^−1^) measured with an osmometer (Osmomat 3000). The chamber was then connected to an alternating power generator (Gerador de Função Digital Minipa MFG-4202, Minipa do Brasil Ltda) operating at 1 V and frequency of 10 Hz for 3 h.

The GUVs were evaluated under an inverted fluorescence microscope (Nikon Eclipse Ti-S) equipped with a 40× objective using excitation (515 to 575 nm) and emission (50 to 80 nm) filters. The images obtained with the Nikon NIS-Elements AR Software (version 3.0) were subsequently evaluated using the Image J program.

### Oocyte collection and maturation

Cumulus-oocyte complexes (COCs) were obtained from cattle ovaries collected at a local abattoir by aspirating antral follicles (3–8 mm in diameter) with an 18-gauge needle attached to a 10-mL syringe. COCs with at least four layers of cumulus cells with homogenous cytoplasm were selected for the experiments. The selected COCs were washed and cultured in 500 μL of IVM medium (30–40 oocytes per well) in a 4-well dish (Nunc, Nunclon Delta Treated 4-Well IVF Dish, Denmark), without mineral oil covering, for 22 h, at 38.5 °C under an atmosphere of 5% CO_2_ in air at maximum humidity.

### In vitro fertilization and embryo culture

Following IVM, oocytes were cultured for 18 h in 500 μL IVF-TALP containing spermatozoa. Motile sperm were obtained by centrifuging frozen-thawed semen through a discontinuous Percoll (GE Healthcare, Uppsala, Sweden) density gradient (45% over 90%) at 2,500 g for 5 min at room temperature. Sperm were diluted in IVF-TALP to achieve a final concentration of 1 × 10^6^/mL in the fertilization well. After removal of cumulus cells, the presumptive zygotes were transferred to 500 μL mSOF medium (25–30 zygotes per well) in a 4-well plate, without mineral oil covering. Embryos were cultured at 38.5 °C in a humidified atmosphere of 5% CO_2_ in air for up to 7 days. The day of in vitro fertilization (IVF) was defined as day 0 (D0). The cleavage rate was assessed at 72 h post-insemination (D3) and the blastocyst rate at 168 h post-insemination (D7). The D7 blastocyst rate was determined based on the number of inseminated oocytes.

### Oxidative stress detection

Intracellular ROS levels in D7 blastocysts were quantified using a fluorescent probe (CellROX Green Reagent, Thermo Fisher Scientific, Waltham, MA, USA). The embryos were stained for 30 min in the dark at 38.5 °C in an atmosphere of 5% CO_2_ in air. Next, the embryos were washed twice in PBS, fixed with 4% paraformaldehyde for 15 min, and washed again with PBS. The embryos were permeabilized with 0.5% Triton X-100 in 0.1% sodium citrate for 10 min. The DNA of the embryos was stained with Hoechst 33342 (1 μg/mL) for 15 min. Stained embryos were washed twice with PBS and observed immediately with a 40× objective under an Olympus IX51 inverted microscope running the Q-Capture Pro Image software (Media Cybernetics, Inc., Version 5.0.1.26) at excitation and emission wavelengths, respectively, of 495 and 520 nm (green emission filter) and 404 and 526 nm (blue emission filter) to quantify the fluorescence signal intensities (pixels). The background signal intensity was subtracted from the measured values of the treated photomicrographs. For each embryo, the CellROX staining intensity was normalized to the DNA. The mean relative expression values were expressed as arbitrary units of fluorescence (AUF).

## Experimental design

### Experiment 1: effect of menadione on oxidative stress induction in in vitro cultured GUVs

GUVs were electroformed and diluted in mSOF (20%, v/v). From this solution, 500 μL was transferred to 4-well plates and increasing concentrations of menadione (1.0, 2.5, 5.0, and 7.5 µM) were added to each well. In the control group, GUVs were cultured in the absence of menadione. The plates were incubated for up to 168 h at 38.5 °C in a 5% CO_2_ atmosphere at maximum humidity. The diameter (µm) of GUVs was measured at the beginning of culture (D1) and after 72 (D3) and 168 h (D7) of culture.

### Experiment 2: effect of hydrogen peroxide on oxidative stress induction in in vitro cultured GUVs

GUVs were electroformed and diluted in mSOF (20%, v/v), as described for Experiment 1. From this solution, 500 μL was transferred to 4-well plates and increasing concentrations of H_2_O_2_ (0.01, 0.05, 0.1, 0.5, and 1.0 mM) were added to each well. The plates were incubated for up to 168 h at 38.5 °C in a 5% CO_2_ atmosphere at maximum humidity. The diameter (µm) of GUVs was measured at the beginning of culture (D1) and after 72 (D3) and 168 h (D7) of culture.

### Experiment 3: induction of oxidative stress by menadione in embryos cultured in vitro in the presence of GUVs

In the first series of this experiment, presumptive zygotes produced in vitro were removed from the fertilization droplets on D1, washed, and transferred in groups of 25–30 zygotes per well to 4-well plates in 500 μL mSOF without (control, *n* = 219) or with 5.0 μM menadione supplementation from D6 to D7 (MD 5.0 μM, *n* = 172). The embryos were cultured for 7 days and the expanded D7 blastocysts were stained for oxidative stress detection.

In another experiment, the presumptive zygotes were removed from the fertilization droplets on D1, washed, and transferred in groups of 25–30 zygotes per well to 4-well plates in 500 μL mSOF according to the following treatments: (1) control (*n* = 100): embryos cultured in mSOF; (2) GUV (*n* = 104): embryos cultured in mSOF plus 20% (v/v) GUVs; (3) GUV + MD 5.0 μM (*n* = 104): embryos cultured as described for the GUV group and supplemented with 5.0 μM menadione from D6 to D7, and 4) GUV + MD 7.5 μM (*n* = 100): embryos cultured as described for the GUV group and supplemented with 7.5 μM menadione from D6 to D7^39^. The embryos were cultured for 7 days without medium changes (feeding) during culture since one of the objectives of this experiment was to evaluate the morphometry and proportion of remnant GUVs at the end of culture. The diameter and quantity (expressed as percentage) of GUVs were evaluated at the beginning (D1) and end of culture (D7). The percentage of GUVs on D7 was determined in relation to the total number of GUVs present on D1. Blastocysts obtained on D7 were evaluated regarding intracellular ROS levels. The culture system was defined as embryotoxic when there was a significant decrease in the blastocyst rate compared to control.

### Experiment 4: evaluation of the effectiveness of GUVs as cysteine carriers in in vitro culture medium of bovine embryos

Presumptive zygotes were transferred in groups of 25–30 zygotes per well to 4-well plates in 500 μL mSOF and cultured according to the following treatments: (1) control (*n* = 111): culture in mSOF; (2) Cyst (*n* = 112): culture in mSOF supplemented with 0.6 mM cysteine from D1 to D7; (3) MD (*n* = 111): culture in mSOF supplemented with 5.0 μM menadione from D6 to D7; (4) MD + Cyst (*n* = 110): culture in mSOF supplemented with 0.6 mM cysteine from D1 to D7 and with 5.0 μM menadione from D6 to D7; (5) GUV(Cyst 3 mM) (*n* = 116): culture in mSOF plus 20% (v/v) GUVs electroformed with 3 mM cysteine (from D1 to D7); (6) GUV(Cyst 3 mM) + MD (*n* = 113): culture in mSOF plus 20% (v/v) GUVs electroformed with 3 mM cysteine (from D1 to D7) and supplemented with 5.0 μM menadione from D6 to D7. The embryos were cultured at 38.5 °C in a 5% CO_2_ atmosphere in air at maximum humidity for up to 7 days. Since one of the objectives of this experiment was to evaluate the morphometry and proportion of remnant GUVs at the end of culture, no medium changes (feeding) were performed during the culture period. Blastocysts obtained on D7 were evaluated regarding intracellular ROS levels.

### Statistical analysis

The experiment was repeated at least three times for each proposed evaluation. The data were analyzed by ANOVA using the JMP 5.0.1a software (SAS Institute, Inc., Cary, NC, USA). Percentage data were arcsine transformed before being submitted to ANOVA when necessary. If a statistically significant effect was found, means were compared by Tukey’s multiple comparisons test. Differences with probabilities (*P*) less than 0.05 were considered to be significant. Values are reported as the mean ± standard error of the mean (SEM).

## Data Availability

All data generated or analyzed during this study are included in this published article.
